# Bio‐Inspired Gradient Coatings Enhance the Leading Edge Erosion Resistance of Wind Blades

**DOI:** 10.1002/advs.77056

**Published:** 2026-08-06

**Authors:** Natalia Sofia Guevara‐Sotelo, Julie Teuwen, Kunal Masania

**Affiliations:** ^1^ Shaping Matter Lab Faculty of Aerospace Engineering Delft University of Technology Delft Netherlands; ^2^ Faculty of Aerospace Engineering Delft University of Technology Delft Netherlands

**Keywords:** bio‐inspired structures, damping, impedance mismatch, leading edge erosion, nacre‐like composites

## Abstract

Rain‐induced erosion of wind blades is a challenge to wind energy growth. As blade lengths and tip speeds increase, droplet‐impact kinetic energy increases, accelerating surface degradation and reducing aerodynamic efficiency. Conventional polyurethane coatings require maintenance and are unable to withstand prolonged exposure to high‐frequency impact stresses. Recent approaches have investigated impedance‐matched multilayer and particle‐reinforced coatings, but these often suffer from abrupt impedance transitions and weak interfacial adhesion. Here, we demonstrate that a bio‐inspired, platelet‐reinforced polyurethane coating with a graded through‐thickness architecture enhances erosion resistance. We reason that minimising the acoustic impedance mismatch between the coating and substrate while maintaining a compliant outer layer reduces interfacial stresses. Compared to monolayer coatings, our system doubles the incubation time under erosion testing, confirming increased durability. Dynamic Mechanical Analysis shows that platelet volume fraction governs the viscoelastic and acoustic impedance behavior, while orientation has negligible influence on viscoelasticity but is critical for wave propagation and damage evolution. We demonstrate that these graded architectures inspired by natural impact‐resistant structures offer superior protection. By providing a deeper understanding of the interplay between acoustic impedance, viscoelasticity, and wave propagation, our study lays the groundwork for designing bio‐inspired graded coatings that actively mitigate impact damage in renewable energy applications.

## Graphical abstract

Rain‐induced erosion of the wind turbine blade leading edge poses a critical challenge to maintenance and operation. Here, we propose a bio‐inspired approach for erosion protection utilising a platelet‐reinforced polyurethane coating with a graded through‐thickness architecture. This approach doubles the erosion resistance time by minimising the acoustic impedance between the different layers.

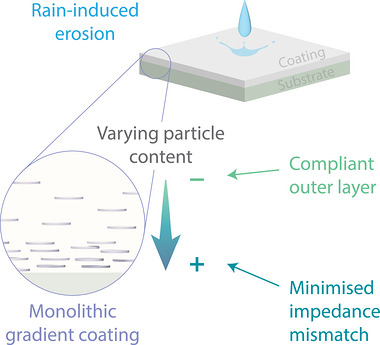

## 1 Introduction

To achieve current global energy targets that aim for a carbon‐neutral system by 2050, the rapid development of wind energy is key. This acceleration has led to a significant increase in wind turbine blade size, with modern blades exceeding 130 meters in length [1]. Although this blade length increase maximizes the energy produced, it also increases the speed at the tip of the blade. Tip speeds can surpass 100 m/s [2], which significantly amplifies the kinetic energy of rain droplet impacts on the leading edge. These high impact energies accelerate surface deterioration, resulting in damage known as leading edge erosion. This damage initiates with micro‐pitting of the blade coating and evolves into severe material loss, compromising the blade's aerodynamic profile and reducing lift while increasing drag. This reduces the performance of the wind turbine, as even moderate erosion can reduce annual energy production by 2%–5%, with severe cases reaching losses of up to 8% [3, 4]. The performance losses not only affect individual turbines but also propagate through wind farms by altering wake dynamics, further decreasing energy production [5]. The urgency to deepen the understanding of the underlying physical mechanisms that govern erosion phenomena is high.

Leading edge erosion is a complex, interdisciplinary phenomenon governed by the interaction of multiple physical mechanisms, including wave propagation and material viscoelasticity. Upon droplet impact, stress waves are generated and propagate through the coating and substrate system. The ability of a material to dissipate these stress waves is largely influenced by its viscoelastic properties, which determine the damping behavior and energy dissipation capacity [4]. Materials with high loss factors, such as certain polyurethane elastomers, exhibit superior erosion resistance due to their ability to attenuate stress waves through internal friction and delayed elastic response [6]. For optimal erosion resistance, coatings should exhibit a balanced viscoelastic profile, where the storage modulus is sufficiently high to maintain structural integrity under impact, while the loss factor (*tanδ*) remains elevated to enable effective energy dissipation [7]. Studies have shown that polyurethane coatings with moderate storage moduli (in the range of 10–100 MPa) and high loss factors (> 0.3) at frequencies relevant to the leading edge erosion regime [8, 9] perform best under rain erosion conditions, as they combine elasticity with damping capacity [7, 10, 11, 12]. The leading edge erosion performance also depends on the material's resistance to crack initiation and growth, since damage progresses through delaminations at weak interfaces between coating layers or between the coating and substrate. Consequently, sufficient bulk strength and interfacial fracture toughness are needed alongside a favorable viscoelastic profile to prevent debonding and subsurface crack propagation under cyclic droplet impact. Despite achieving a favorable balance of material properties, these typical coatings still require regular maintenance every 3–5 years, and therefore, there is a need to implement other coating technologies, such as impedance‐matched layers and particle‐reinforced coatings.

Recent advances in coating technologies demonstrate a shift toward more resilient materials specifically designed to counteract the physical effects that occur during erosion. One design strategy that has gained increased attention is the concept of acoustic impedance‐matching layers, which minimize wave reflection and enhance stress transmission at material interfaces [13]. By minimising this impedance mismatch between the layers and the substrate, the extent of erosion damage and adhesion at the coating‐substrate interface is improved [6], thus increasing the coating's lifetime. It is demonstrated that, by minimising the acoustic impedance between layers, coatings can become more resistant to rain erosion, with multilayer systems offering improved energy dissipation and reduced surface degradation [11, 14]. Typical multilayer systems consist of filler, primer, and topcoat layers, each contributing to the overall mechanical and acoustic behavior [13]. However, the interface between layers is a critical zone where delamination can occur if impedance mismatches are large and adhesion is poor. In commercial leading edge protection systems, putty layers are added beneath the main outer polyurethane topcoat to address this transition. They are formulated to provide a gradual acoustic bridge between the stiff composite substrate and the more compliant coating, reducing abrupt impedance steps and attenuating stress reflections at the coating–substrate interface. This highlights the importance of controlled impedance gradients and the need for more engineered coating architectures. To further enhance the mechanical robustness of these multilayer systems, researchers are increasingly exploring the integration of additional strategies to dissipate energy more effectively within the coating system.

An approach that has emerged in coating development is the implementation of nano‐ or micro‐particle reinforced coatings to mitigate incoming stress waves. Initial studies on reinforced coatings have demonstrated improved wear resistance [15], hydrophobicity [16], loss factor in laboratory tests [17], and tensile, compression, and tearing mechanical testing [18]. Further research demonstrated improved solid‐particle erosion with the implementation of reinforcing particles made of cellulose [19], graphene [18, 20, 21], ceramic oxides like aluminium and silica oxide [22, 23], and epoxy‐terminated polysulphide [24]. Grundwürmer et al. [25] applied a stationary sample erosion test, where a high‐pressure water jet repeatedly moved across coated aluminium samples with nano‐silica and nano‐alumina reinforcement, simulating droplet impacts at velocities up to 350 m/s. The number of jet passes until visible damage was reported as a measure of erosion resistance. Their results demonstrated improved erosion performance for some of the particle‐reinforced coating systems and highlight the importance of selecting a proper reinforcement.

Although particle‐reinforced coatings have shown promise through improved mechanical properties and solid impact testing, their performance under realistic rain erosion conditions remains insufficiently explored. Many studies rely on indirect metrics, such as solid particle erosion or low‐frequency viscoelasticity, which do not fully capture the dynamic stress wave interactions relevant in leading edge erosion scenarios on wind turbine installations. Further, structural gradients in coatings have been proposed to enhance impact absorption [6]; however, their implementation and validation under high‐speed droplet impact remain limited. To advance erosion protection, coatings must be engineered not only for mechanical properties but also for dynamic wave mitigation, which requires a deeper understanding of viscoelastic behavior in the relevant frequency regime.

**FIGURE 1:**
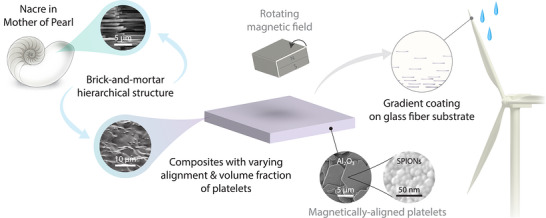
Overview of nacre‐like platelet‐reinforced polyurethane composites for leading‐edge erosion protection of wind turbine blades. The hierarchical brick‐and‐mortar structure of nacre [71] enables it to exhibit high fracture toughness while maintaining its strength and stiffness through localized deformation. These properties can be achieved in polymeric materials by reinforcing them with functionalized platelets. The platelets are electrostatically coated with superparamagnetic iron oxide nanoparticles (SPIONs) to make them magnetically responsive to an external rotating magnetic field and then embedded in polyurethane. The resulting polyurethane‐alumina composites are tested for erosion protection of glass fibre substrates.

We hypothesize that probing the viscoelastic properties and damage mechanisms in strain rates relevant to leading‐edge erosion can lead to the design of dissipative materials as readily found in nature. One such promising example is nacre, found in mother‐of‐pearl. Through hierarchical structuring of weak, brittle building blocks, the material achieves remarkably high stiffness without sacrificing damage tolerance [26, 27, 28]. Beyond this hierarchical arrangement, nacre and many other natural armour systems, such as pomelo peel and insect cuticle, also rely on a gradual, graded variation in stiffness and density through their thickness, rather than an abrupt transition between hard mineralized layers and soft underlying tissue. This graded transition minimizes the acoustic impedance mismatch between adjacent layers, thereby reducing the reflection and localization of stress waves that would otherwise concentrate damage at a sharp interface. Their hierarchical structure inspires us to explore the role of lamellar patterns in coatings to tune the viscoelastic properties and damage mechanisms of wind blade coatings. This can be achieved through the introduction of reinforcing platelets, where the composition and orientation can be precisely tuned via platelet functionalization and magnetic alignment, as shown in Figure 1. We propose a carefully designed bio‐inspired composite coating with a variable through‐the‐thickness platelet content that gradually changes the impedance. We propose that minimising the mismatch between the coating sublayers and the substrate will reduce stress peaks at the interfaces and result in higher incubation times, i.e., better resistance to erosion.

## 2 Results

### 2.1 Erosion Testing of the Platelet‐Reinforced Composites

**FIGURE 2:**
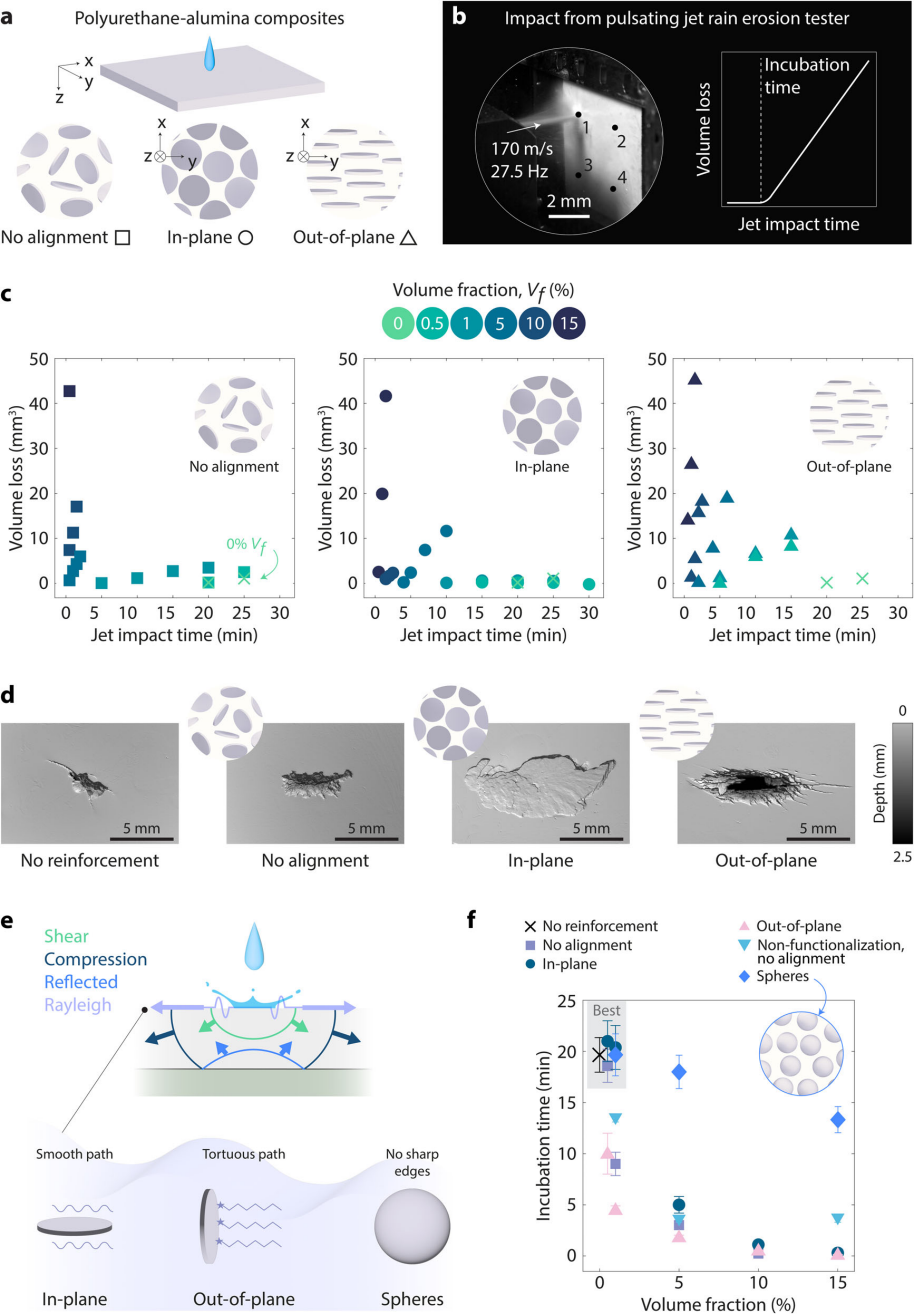
Erosion testing of the composites with different orientations and platelet volume fractions. (a) The platelet orientation (no alignment, in‐plane, and out‐of‐plane alignment) is defined by the droplet impact direction (*z*‐direction). (b) Rain erosion was assessed using a pulsating‐jet erosion tester at a constant impact velocity and frequency. The left image shows this process captured with a high‐speed camera. Each sample underwent up to four tests, where the volume loss and incubation were determined. (c) The volume loss at different jet impact times was determined for composites with no alignment, in‐plane alignment, and out‐of‐plane alignment. (d) The damage morphology was assessed via microscopy and is characteristic of each type of alignment. (e) Wave propagation during liquid‐solid impact is characterized by different wave fronts. From these, we highlight the Rayleigh wave front and how the reinforcement affects it. (f) The incubation time for composites with no alignment, in‐plane alignment, and out‐of‐plane alignment is compared to that of composites with spherical reinforcement and non‐functionalized and non‐aligned platelets.

Polyurethane specimens (Xencast PX60, Easy Composites, United Kingdom) 40 mm ×  40 mm with a nominal thickness of 2.5 mm were reinforced with alumina platelets (White Sapphire, Rona Flair, Merck, Germany) with a nominal diameter of 8.3 µm at different volume fractions and orientations via magnetic alignment [26]. The non‐magnetic alumina platelets were functionalized with superparamagnetic iron oxide nanoparticles (EMG‐705, Ferrotec, United States) to create a thin shell containing 5 nm iron oxide particles on their surface, making the platelets magnetically responsive. These platelets and functionalization methodology were chosen because it follows established prior work by Woigk et al. [26], making our results directly comparable to a validated benchmark that already demonstrated improved damping upon magnetic alignment. Polyurethane‐alumina suspensions are cast in silicone molds under the influence of magnetic fields. When the platelets are exposed to rotating external magnetic fields with a frequency that surpasses a threshold value, viscous forces exerted by the suspending polymer confine and align the platelet motion to the plane of the magnetic field's rotation [26]. Via magnetic alignment, composites with in‐plane alignment and out‐of‐plane alignment were manufactured. This orientation is defined based on the direction of the incoming droplet, as shown in Figure 2a. In addition to the platelet orientation, the volume fraction (*V_f_*) was varied from 0% to 15%. The rain erosion performance of the polyurethane‐alumina composites was studied using a pulsating‐jet rain erosion tester (PJET, Ducom Instruments, India). The PJET ejects water through a nozzle at a controlled velocity of 170 m · s^−1^ to generate discrete water slug impacts at a frequency of 27.5 Hz on a stationary specimen, simulating repeated raindrop impacts as shown in Figure 2b. At these testing conditions, each slug contains approximately 150 mm^3^ of water. For each sample, four impact points were used, where the jet impact time was increased to study the volume loss after a given jet impact time. The volume loss was determined using a wide‐area microscope (VR‐5000, Keyence, Japan) to determine the incubation time. The value of incubation time is determined at the moment at which volume loss is first observed to evaluate the performance of coatings used to prevent leading edge erosion [29].

Figure 2c illustrates the evolution of volume loss over time for composites with no alignment, in‐plane alignment, and out‐of‐plane alignment, highlighting that increasing the platelet volume fraction consistently accelerates erosion, as evidenced by steeper loss rates and shorter incubation periods. This behavior is in accordance with previous findings that incubation time inversely correlates with coating stiffness and filler content. This can be reasoned because harder surfaces dissipate less impact energy elastically, leading to higher stress concentrations during droplet impact [30, 31]. Ceramic platelets significantly increase the hardness and modulus of the polymer matrix [32], which, while advantageous for wear resistance, paradoxically promotes faster liquid impact damage because harder coatings facilitate more pronounced lateral jetting and therefore more transverse shear stresses [31]. When platelets are oriented with out‐of‐plane alignment and facing the incoming droplet, we observe the steepest erosion rates, followed by random and in‐plane alignment. This is consistent with fracture mechanics observations that platelet edges act as stress concentrators when aligned perpendicular to the surface [33, 34]. This anisotropy mirrors trends in platelet‐reinforced ceramics and nacre‐inspired composites, where orientation governs crack deflection and energy dissipation pathways [26]. To understand these mechanisms, damage morphology must be examined, as prior work on particle‐reinforced systems shows the relevance of reinforcement, microcrack initiation, interfacial debonding, and platelet fracture dominate early‐stage erosion [35].

The impact of platelet orientation on damage is visualized in Figure 2d, where the distinctive damage for each type of architecture is compared with representative microscopy images. The damage morphology reveals that the microstructural architecture is reflected at the macroscopic scale. Samples with in‐plane orientation show shallow and “crater‐like” damage that occurs in layers, whereas samples with out‐of‐plane orientation present damage in the form of thin and long incisions that penetrate the whole thickness. This morphology is consistent for all in‐plane and out‐of‐plane composites. However, the size and depth of it strongly depend on the volume fraction, as can be seen in the full erosion maps presented in Figures S1–S3. Samples with no alignment exhibit less consistent damage morphology, which is dominated by the volume fraction. The test results in a characteristic damage shape in the longitudinal direction. This might be related to the rotation of the disc, which controls the ejection of water droplets. The rotating disc consists of a plate with two holes that uncover the front of the nozzle for a calculated period of approximately 0.4 microseconds. This causes water from the nozzle to be ejected in a left‐to‐right direction, explaining the longitudinal shape of the damage, which can be verified by observing the unreinforced samples. We highlight that the role of water jetting on the damage phenomena seems to be significant. Lebar et al. [36] conducted a study in which they tested the spall strength using solid projectile sheets at high velocities. They compared the damage morphology of polyurethane with alumina spheres and irregularly shaped alumina inclusions. In their work, the damage morphology remained unchanged for the different shapes of inclusions. This further supports the role of droplet deformation in introducing stress to the coating during experimentally simulated leading edge erosion. We hypothesize that the reason behind this distinctive damage morphology for in‐plane and out‐of‐plane platelets is related to the mechanics of wave propagation during liquid‐solid impact.

Wave propagation during leading edge erosion can be described in different stages with distinctive wavefronts, as shown in Figure 2e. The initial stage begins when a raindrop impacts the leading edge at a high speed, generating a water‐hammer pressure pulse within a few nanoseconds [37, 38]. This sudden pressure peak launches longitudinal compression waves into the coating and substrate, travelling throughout the material at a velocity related to the dynamic through‐thickness elastic modulus of the coating layers and substrate, and their impedance mismatch to each other. Part of this compressive pulse is then reflected and converted at the various material interfaces (coating‐coating or coating–substrate), resulting in transverse shear waves and surface Rayleigh waves that travel along the coating surface [4, 38, 39]. When these waves travel through typical viscoelastic materials, they attenuate and interact, resulting in complex stress fields that are strongly influenced by the acoustic impedance mismatch between layers. In the third stage, the Rayleigh surface wave dominates the stress field near the surface, causing tensile stresses within the coating surface that initiate damage. Simultaneously, the acceleration resulting from droplet impact creates a lateral jet that exerts additional stresses on the coating surface, further interacting with the propagating surface Rayleigh waves. The behavior of these wave fronts governs how energy is distributed through the coating, making wave propagation a critical mechanism in understanding leading‐edge erosion phenomena.

During wave propagation, reflections and impedance mismatches occur between the polymer and the platelets. The more platelets, the more reflections we will have, which cause stresses that result in earlier damage compared to the unreinforced material. The platelets act as small stress concentrators, and if the platelets are oriented in the out‐of‐plane direction, the sharper edge of the platelets will result in a higher stress concentration factor in the tensile stress region of the coating, leading to accelerated damage. From a wave propagation perspective, the Rayleigh surface wave is considered the most influential front driving leading edge erosion. After the initial water‐hammer pressure launches longitudinal compression and transverse shear waves, the Rayleigh wave propagates along the surface of the coating. This concentrates tensile and shear stresses within the top tens of micrometers of the coating, where microcracks initiate and grow [40, 41, 42]. Compared to the platelet average diameter of 8.3 µm, this distance from the surface, where stresses concentrate, is of the same order of magnitude and therefore leads to damage onset at lower stresses. If platelets are out of plane, they may create a tortuous path for the waves to travel, as more obstacles are encountered. However, this is less the case with in‐plane alignment, which offers a relatively smoother propagation path and lower stress amplification. When platelets are not deliberately aligned, the coating naturally exhibits a combination of in‐plane and out‐of‐plane orientations. This hybrid arrangement creates a balance between the two extremes: it avoids the severe stress concentration and tortuous wave paths associated with fully out‐of‐plane alignment, while also sacrificing some of the smooth propagation benefits of fully in‐plane alignment. This behavior is analogous to fibre‐reinforced polymers, where fibre orientation strongly influences crack initiation and propagation under dynamic loading [43, 44]. To further isolate the effect of filler volume fraction from geometric factors, we hypothesize that by changing to spheres, we can reduce the stress concentrations observed due to the sharp edges of the platelets, thereby reducing damage and isolating the effect of filler volume fraction from aspect ratio.

We validated this hypothesis by comparing the incubation times of platelet‐reinforced composites with those reinforced by spherical inclusions, as shown in Figure 2f. Composites containing 1%, 5%, and 15% sphere volume fractions were tested under identical conditions. The results confirm that changing the filler geometry from platelets to spheres of comparable size and stiffness (both alumina) has a significant influence on wave propagation and damage evolution. The spherical shape reduces stress concentrations at the filler–polymer interface, leading to markedly higher incubation times. This effect becomes dominant at higher volume fractions (5% and 15%), where the improvement exceeds threefold compared to platelet‐reinforced coatings across all alignments. Additionally, we tested whether functionalization influenced the erosion performance by manufacturing composites with platelet volume fractions of 1%, 5%, and 15% without any prior treatment. Non‐functionalized platelets exhibited better erosion performance than those with functionalized non‐aligned platelets, likely due to improved adhesion with the polyurethane. Nanoparticle coverage was not directly measured, but based on the ferrofluid concentration used, the resulting iron oxide coverage falls within the low volume fraction range (0.01%–1 vol%) reported by Erb et al. for magnetic functionalization [45], suggesting sparse rather than continuous surface coverage. Even at this low coverage, the nanoparticles preferentially occupy the reactive Al–OH sites that otherwise bond with the polyurethane, so adhesion decreases disproportionately relative to the small amount of iron oxide added. Optimising interfacial bonding remains critical, and future work should explore strategies to enhance compatibility without compromising erosion resistance, for example, by using silane treatment [36].

The effect of platelet orientation on erosion rate is most pronounced at lower volume fractions, where alignment dictates stress wave propagation and damage onset. As the volume fraction increases, the dominant failure mechanism shifts toward stress concentrations caused by the higher platelet content and the greater probability of local failures. This contrasts with lower volume fractions, where orientation effects prevail. Our results confirm that both the reinforcement shape and its orientation relative to droplet impact play a critical role in determining erosion performance. Literature further supports that filler addition and platelet orientation influence key viscoelastic parameters, namely storage modulus, loss modulus, and loss factor, all of which govern a material's ability to dissipate impact energy. To quantify these effects and link reinforcement to mechanical behavior, we performed dynamic mechanical analysis.

### 2.2 Understanding the Role of Platelet Reinforcement With Dynamic Mechanical Analysis

**FIGURE 3:**
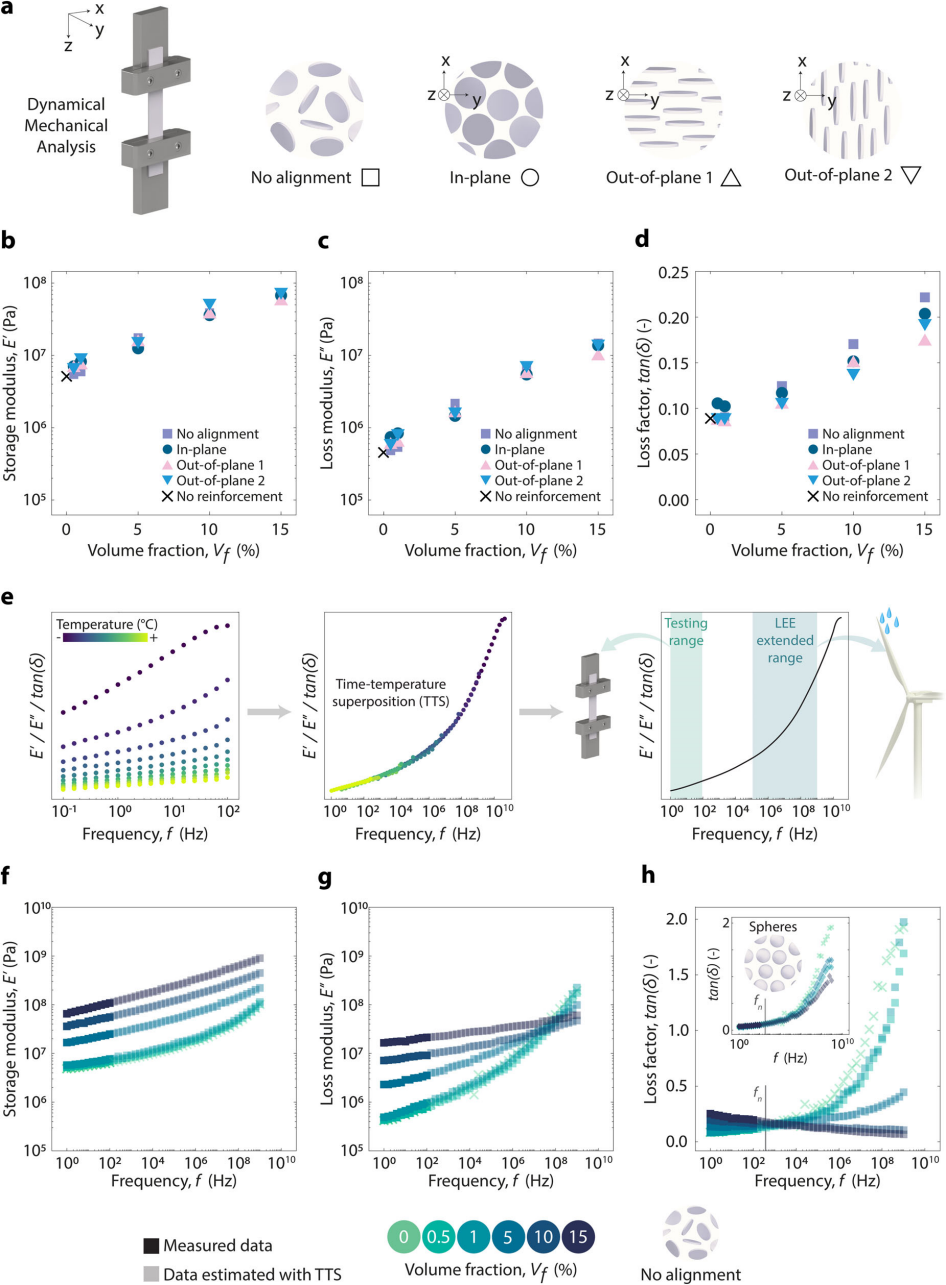
Dynamical Mechanical Analysis (DMA) of the composites with different orientations and platelet volume fraction. (a) The platelet orientation is relative to the direction of the applied tensile force (*z*‐direction). (b) The storage modulus (*E′*), (c) Loss modulus (*E″*), and (d) Loss factor (*tanδ*) are shown for composites with no alignment, in‐plane alignment, and out‐of‐plane alignment at an oscillatory frequency of 10 Hz and a temperature of 30°C. (e) A time‐temperature superposition (TTS) was implemented to extend the frequency range to that occurring during droplet impact on the leading edge at a reference temperature of 30°C. (f) The storage modulus (*E′*) and (g) Loss modulus (*E″*) master curves obtained from the TTS are presented for composites with no alignment. (h) The loss factor (*tanδ*) is presented for composites with no alignment and spherical reinforcement. The estimated natural frequency (*f_n_*) of the near polyurethane (343 Hz) is highlighted.

To investigate how platelet orientation and volume fraction influence the viscoelastic response of the composites, an oscillatory loading was used under Dynamic Mechanical Analysis (DMA) to quantify the storage modulus (*E′*), loss modulus (*E″*), and loss factor (*tan δ*). Composites with 0% to 15% platelet volume fraction were tested in tension, with the orientation defined with respect to the loading direction, as illustrated in Figure 3a. To benchmark the effect of platelet alignment and give an initial comparison with existing literature on nacre‐inspired systems, we focused our initial analysis on *E′*, *E″*, and *tan δ* at 10 Hz and 30°C.

The storage modulus, loss modulus, and loss factor all increase consistently with platelet volume fraction, as shown in Figure 3b–d. This trend reflects the formation of regions in the material that restrict polymer chain mobility, thereby increasing stiffness, while simultaneously introducing more interfacial regions that contribute to energy dissipation, as evidenced by the rise in loss modulus [46, 47]. Calculations show that we fall at values far below the percolation threshold, so the material does not experience stress transfer through platelet‐continuous phases, but rather through shear lag between the platelets and the polymer. The corresponding increase in *tanδ*, combined with the percolation concentration below, suggests that damping at the tested frequency is enhanced by interfacial friction and polymer–filler interactions rather than platelet orientation, a conclusion supported by Libanori et al. [47], who demonstrated that hierarchical reinforcement of polyurethane with micro‐ and nanoplatelets enhances stiffness without compromising damping. Furthermore, the absence of significant differences between in‐plane and out‐of‐plane alignment suggests that platelet alignment does not influence the viscoelastic response under the tested conditions. The relationship between *tan δ* and volume fraction aligns with the surface‐area‐controlled damping hypothesis, where increasing platelet surface area amplifies polyurethane–platelet interactions and interfacial frictional losses [46]. Collectively, these trends point to interphase and segment‐level mechanisms in segmented polyurethane that can simultaneously raise *E′* and *E″* without revealing orientation sensitivity.

Segmented polyurethane consists of hard segments, which act as physical cross‐links, and soft segments, which primarily govern viscoelastic deformation. Microphase separation between these domains and hydrogen bonding within the hard segments control both modulus and energy dissipation [48, 49]. When rigid platelets are introduced, they restrict the mobility of adjacent hard segments and increase local cross‐link density, thereby raising the storage modulus (*E′*) [47, 50]. At the same time, higher platelet content creates more polymer–filler interfaces where chains remain partially mobile, enabling frictional sliding and shearing that elevate the loss modulus (*E″*) and loss factor (*tan δ*), a mechanism consistent with surface‐area‐driven damping observed in filled elastomers [46]. Platelet orientation does not seem to influence this response. A likely explanation could be because energy dissipation is dominated by segmental relaxations of the soft domains and distributed interfacial losses around platelet edges [51]. The polyurethane used in this study is estimated to contain 20–40 wt.% hard segments. This range was defined based on reported relationships between hardness and the amount of soft segments [52]. The hard domains form at a much smaller length scale than the platelets (∼8.3 µm), which span across multiple microphase‐separated regions. As a result, the platelets are unlikely to selectively associate with the hard segments, unlike the behavior observed by Libanori et al. [47], where much smaller platelets could effectively reinforce the hard domains. Instead, the platelets interact with a mixed environment of soft and hard segments, and only a thin interfacial layer of the surrounding polymer is dynamically affected, limiting the extent of selective reinforcement or alignment. This behavior contrasts sharply with other polymers such as epoxy and Poly(methyl methacrylate) (PMMA), where the absence of soft segments and the glassy nature of the matrix make orientation effects far more pronounced.

Polyurethane behaves as a rubbery material at the tested temperature of 30°C, exhibiting high segmental mobility. Adding platelets drastically constrains these soft‐segment relaxations, resulting in a 13‐fold higher storage modulus (*E′*) and a 32‐fold higher loss modulus (*E″*) compared to epoxy systems at the same tested frequency of 10 Hz. These large relative gains occur because polyurethane starts from a low‐modulus baseline and benefits from interfacial friction and microphase reinforcement, whereas epoxy's glassy matrix limits such effects. In glassy epoxy at room temperature, chain mobility is minimal, so platelet alignment becomes critical for stress transfer. Nacre‐like architectures in epoxy create tension–shear chains that efficiently transmit loads along aligned platelets, as demonstrated by Woigk et al., who reported up to a 4.5‐fold increase in stiffness while maintaining damping [26, 53]. This behavior approaches that of natural nacre, though nacre is even more extreme: its high mineral content and bridging structures channel stress almost exclusively through stiff CaCO_3_ platelets. Remarkably, nacre exhibits negligible frequency dependence (Figure S4), which is typical of bulk ceramic materials, yet achieves high damping and toughness by partially demineralising and introducing hierarchically organized layers. While nacre represents the upper bound of stiffness–damping, polymer‐based systems offer a different pathway: instead of mineral bridges, they rely on chain mobility and interfacial friction to dissipate energy. This was also examined by Woigk et al. [26] with a methacrylate copolymer, which is more dissipative and less cross‐linked than epoxy but still glassy at room temperature. The storage modulus increased with platelet content and remained like that of the epoxy. However, the loss modulus did not increase as much because it is governed by the matrix's internal molecular relaxation processes. In the case of PMMA, the enhanced chain mobility associated with internal relaxation processes significantly increases the energy dissipation capacity, but it reaches a saturation point relative to the reinforcement level.

To further understand the response upon dynamic loading of our composites within the context of leading edge erosion, we applied a time‐temperature superposition (TTS), as visualized in Figure 3e. TTS is a fundamental principle in polymer viscoelasticity that exploits the equivalence between temperature and time scales in viscoelastic materials to predict the dynamic response beyond the experimental frequency range [54]. We used the frequency sweep over the full capable 10^−1^ – 10^2^ Hz range of our testing equipment at different temperatures. Based on the principle that a temperature change produces an equivalent shift in relaxation times [55], we can shift the frequency‐dependent moduli and obtain a master curve that extends over several decades.

By applying this method, we can obtain the expected response of our composites in the frequency range relevant to leading edge erosion. The frequency range can be estimated based on the timescales that occur during wave propagation phenomena triggered by the impact of liquid droplets. The impact of a typical rain droplet at high speed will cause the initial water hammer pressure to rise within microseconds [37], corresponding to effective frequencies in the kHz—MHz range [37, 56]. Furthermore, Rayleigh surface stress waves, which propagate at velocities near the material's shear wave speed and penetrate to a depth of micrometers, travel in the 10^5^ – 10^7^ Hz regime [39]. Studies that relate the leading edge erosion timescales follow a more conservative approach, extending the frequency range to up to 10^9^ Hz [7, 8]. Considering these estimations, we define the relevant range as between 10^5^ and 10^9^ Hz.

We performed TTS on the DMA results from composites with four different alignments. Figure 3f–h show the master curves for the storage modulus, loss modulus, and loss factor for composites with no alignment. As was the case for a benchmark frequency of 10 Hz, shown in Figure 3b–d, no significant differences were observed between the master curves for different alignments at the same platelet volume fraction. We also performed DMA on composites with non‐functionalized platelets, where no differences were observed. The master curves for composites with in‐plane alignment, out‐of‐plane alignment, and without functionalization are presented in Figures S5–S9. Figure 3f–h indicate the distinction between the measured data and the estimated data, as the TTS for composites with 10% and 15% volume fraction was not validated (Figures S10–S12 and Tables S1 and S2).

The storage modulus (Figure 3f) and loss modulus (Figure 3g) increase consistently with frequency across all platelet volume fractions and different orientations (Figures S5–S7). At low platelet content (0% – 1%), the polyurethane matrix dominates the response: soft segments remain highly mobile at low frequencies but become progressively restricted as oscillation rate rises, producing a steep increase in storage modulus as the material transitions toward glassy‐like behavior. Hard domains, which mechanically act as physical cross‐links, remain relatively insensitive to frequency yet constrain the mobility of soft segments, amplifying this stiffening effect [57]. This frequency sensitivity reflects matrix‐driven mechanisms rather than platelet‐controlled effects, as platelet–polyurethane interfaces are minimal at low volume fractions. The restriction of motion at higher frequencies causes more internal friction, resulting in a sharp increase in energy dissipation. This is reflected by the steep slope of the loss modulus master curve, as frictional constraints convert mechanical energy into heat during rapid oscillations. When the platelet content exceeds 1%, the platelets and their interfacial regions constrain polymer motion, even at low frequencies, thereby raising the storage modulus but reducing the frequency‐induced stiffening. This explains why the master curves flatten compared to low *V_f_* composites. Loss modulus is dominated by localized soft‐segment motion and interfacial friction, both of which are less sensitive to oscillation rate, resulting in a pronounced flattening of the loss modulus curve as well. Consequently, high *V_f_* composites cannot gain significant additional energy dissipation. In contrast, low *V_f_* ones still exhibit frictional motions, causing the loss factor master curves to intersect and reorder, marking a transition from segmental relaxation to platelet‐polyurethane friction‐driven mechanisms.

The loss factor (Figure 3h) represents the ratio of energy dissipated to energy stored and mirrors the interplay between stiffness and damping mechanisms observed in the storage and loss moduli. At low frequencies, high *V_f_* composites exhibit elevated *tan δ*, compared to low *V_f_* ones, due to dominant interfacial friction and constrained polymer motion, consistent with their higher *E″* and moderate *E′*. As frequency increases, low *V_f_* composites show a steep rise in both *E′* and *E″*, but the relative increase in *E″* outpaces that of *E′*, producing a steeper growth of *tan δ*. This behavior reflects the matrix's transition toward glassy‐like dynamics, where segmental motion drives internal friction and high‐frequency damping [51]. In contrast, high *V_f_* composites display a more gradual increase in *E′* and *E″* because platelet–polyurethane interfacial regions, which dominate damping at low frequencies, do not scale with oscillation rate as segment‐driven mechanisms do. Consequently, *tan δ* declines, indicating that energy storage begins to outweigh dissipation and that segmental motion damping saturates. The crossover point, where low *V_f_* composites dissipate more energy than high *V_f_* ones, occurs near the estimated natural frequency of neat polyurethane, marking a shift from platelet‐dominated to matrix‐dominated dissipation mechanisms, a trend confirmed in an alternative polyurethane system (Figure S13). This characteristic behavior is absent in composites reinforced with spheres, which provide lower interfacial friction due to their smooth geometry. As a result, spherical fillers do not significantly constrain polymer motion, allowing the matrix to maintain its damping capacity across the frequency range. This prevents saturation of dissipation mechanisms, leading to a consistent increase in *tanδ* with frequency.

The frequency‐dependent viscoelastic behavior revealed by the master curves offers an interesting insight into how these composites respond under the extreme dynamic conditions associated with leading edge erosion, where impact events occur in the 10^5^–10^9^ Hz range. Erosion testing confirms that composites with higher platelet volume fractions fail earlier and more severely than those with lower reinforcement, a trend explained by the significant decrease in *tan δ* within the relevant frequency regime. Since the loss factor governs energy dissipation during droplet impact [8, 58], its reduction at high *V_f_* highlights why erosion performance deteriorates despite increased stiffness. Moreover, DMA results show no measurable differences in storage or loss moduli between platelet orientations, which contrasts with the erosion behavior and reinforces the conclusion that wave propagation, not viscoelastic anisotropy, drives orientation effects. Having clarified the interplay between platelet content, viscoelasticity, and stress‐wave dynamics, we can now leverage these insights to design coatings that actively mitigate impact damage, paving the way for bio‐inspired gradient architectures that combine impedance matching with controlled reinforcement.

### 2.3 Toward a Bio‐Inspired Gradient Coating

**FIGURE 4:**
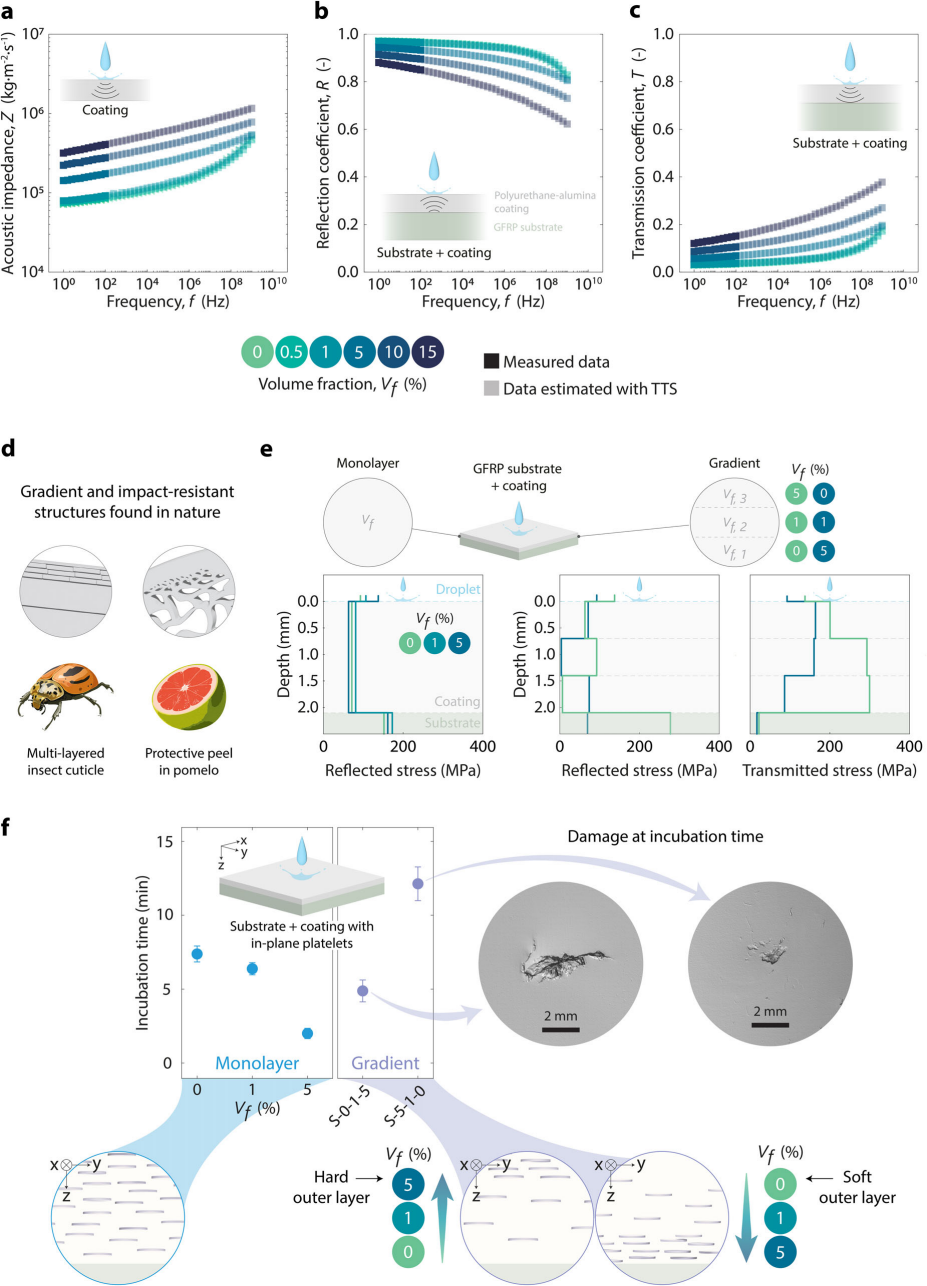
The development of a bio‐inspired gradient coating. (a) The acoustic impedance of composites with no alignment is determined using the DMA and TTS master curves. (b) The reflection and (c) Transmission coefficients are calculated assuming a glass‐fibre‐epoxy substrate with a platelet‐polyurethane coating. (d) Impact‐resistant gradient structures are often found in natural structures. (e) The reflected and transmitted stresses resulting from an incoming rain droplet are calculated for a monolayer and gradient coating on top of a glass‐fibre epoxy substrate. (f) The incubation time for a monolayer and gradient coating on a glass fibre‐epoxy substrate is determined using pulsating‐jet rain erosion testing equipment.

Additional platelet‐dependent phenomena that occur during leading edge erosion can be explained using Dynamic Mechanical Analysis. Building upon the frequency‐dependent behavior of the storage and loss moduli, the acoustic impedance of the composites was evaluated to further understand their dynamic response under high‐frequency oscillations. The acoustic impedance is defined as Z=ρ·c [59], where ρ is the density and *c* is the acoustic velocity. The acoustic velocity is dependent on the complex modulus through the relationship $c\left(\omega \right)=\sqrt{\frac{E^{*}\left(\omega \right)}{\rho }}\;$, where $E^{*}\left(\omega \right)=\sqrt{|E^{\prime }{\left(\omega \right)}^{2}+E^{\prime \prime }{\left(\omega \right)}^{2}|}$ is the magnitude of the complex modulus obtained from Dynamic Mechanical Analysis [60]. This formulation, rather than using the storage modulus *E′* alone, captures the contribution of both the elastic and dissipative components of the viscoelastic material's response to wave propagation and inherently links the impedance to both the stiffness and energy‐damping characteristics of the material. As shown in Figure 4a, the impedance increases with frequency for all platelet volume fractions, consistent with the stiffening and dissipative behavior observed in the storage and loss moduli master curves. However, the rate is strongly dependent on the volume fraction. At low platelet content (0%–1%), the impedance exhibits a steep rise with frequency, reflecting the transition of the polyurethane matrix from a rubbery to a glassy‐like regime. This is driven by segmental motion of the soft domains, which dominate the viscoelastic response at low reinforcement levels. In contrast, composites with platelet volume fractions above 1% show a more gradual increase in impedance, indicating that the matrix mobility is already constrained by the dense platelet network and interfacial friction, which limits further stiffening with increasing strain rate. This behavior mirrors the trends observed in the loss modulus and *tanδ* master curves, where damping mechanisms shift from segmental relaxations to interfacial friction as *V_f_* increases. The reduced sensitivity of impedance to frequency at high *V_f_* suggests that wave propagation becomes increasingly governed by the rigid platelet architecture rather than the polymer matrix. Our findings highlight the role of platelets in tuning both stiffness and acoustic impedance, making them a fascinating controllable design parameter for engineering materials with specific impedance requirements.

The acoustic impedance of our composites can be used to determine the reflection and transmission coefficients when implemented as a coating on a glass fibre‐epoxy substrate with an estimated impedance of 5 MRayl [61], which is typical of the materials used for the blade's leading edge. These coefficients quantify the extent to which the stress wave generated by droplet impact is reflected into the coating or transmitted into the substrate. They are defined as *R* and *T* in the following relationships: $R=\frac{Z_{1}-Z_{2}}{Z_{1}+Z_{2}}\;$, $T=\frac{2Z_{2}}{Z_{1}+Z_{2}}\;$, where *Z*_1_ and *Z*_2_ are the impedances of the incident and transmitted media, respectively [59]. A high reflection coefficient implies that a significant portion of the stress wave remains within the coating, potentially leading to localized damage such as microcracking or surface fatigue. Conversely, a high transmission coefficient indicates that the wave propagates into the substrate, where it can trigger delamination or fibre–matrix debonding, accelerating structural degradation [14]. Although a low transmission coefficient can protect the laminate from deep damage, it concentrates energy in the coating, which may accelerate surface erosion [59]. In the context of leading edge erosion, the coating should ideally exhibit an impedance match that minimizes abrupt changes at the interface, reducing harmful reflections while attenuating transmitted waves through viscoelastic dissipation [11, 14, 59]. This impedance matching is evident in the values of the reflection and transmission coefficients for our composite coating and substrate system, as shown in Figure 4b,c. Because the impedance changes with platelet volume fraction, we observe lower reflection and higher transmission coefficients as the platelet content increases. This dependency opens opportunities to tailor coating performance by adjusting platelet content to control stress wave distribution at the interface, or even multiple interfaces if multiple layers with stiffness gradients are implemented.

Natural impact‐protection structures often present graded architectures (Figure 4d). These architectures provide smooth transitions in stiffness and density, which help mitigate stress from impact forces [62, 63]. Across these layers, a gradual stiffness and, therefore, acoustic impedance gradient facilitate smooth wave transmission and minimize abrupt reflections under impact. In the turtle carapace, a keratinous top layer, a foam‐like cancellous core, and a dense cortical bone form a functionally graded sandwich structure, where the modulus and mineral content decrease from the outer cortex toward the interior. This structure facilitates a gradual redistribution of stress and crack deflection across layers, thereby enhancing impact resistance and preventing catastrophic failure [64]. This same mechanism of protection is observed in insect cuticles, where, instead of relying on porosity changes, three distinctive layers create a gradient where the outer layers are hard and rigid to prevent damage, while the inner layers are soft and compliant to absorb impact and protect internal structures [65]. These graded structures are not exclusive to animals and can be found in nuts and fruits [63]. This is the case for the pomelo fruit, whose peel consists of three distinct layers that collectively create a protective mechanism mitigating mechanical stresses and impact damage to the fruit. The outer layer serves as a dense barrier, the middle sponge‐like layer absorbs and dissipates energy, and the inner layer is loosely arranged to cushion the fruit pulp, ensuring its integrity upon impact. Remarkably, despite the vast differences in size, impact forces, and strain rates, these biological systems converge on a common design strategy for impact tolerance. Although the impact protection requirements for these organisms are fundamentally different from our coating‐substrate system, since the role of the graded structure of the organisms is to protect a soft body, we can apply this concept to engineer a coating for enhanced leading edge erosion protection where the requirement is to protect a hard composite substrate.

Bio‐inspired stiffness and impedance gradients have the potential to improve the erosion performance of materials by matching the interface between the different layers in the system (droplet‐coating, coating‐coating, and coating‐substrate). Here, stress wave transmission becomes more gradual and less disruptive, reducing interfacial reflections and minimising localized stress concentrations that typically lead to delamination of the substrate and surface degradation of the coating under erosive conditions [14]. To demonstrate this capability with our platelet‐reinforced composites, we estimated the reflected and transmitted stresses at the different interfaces resulting from a compressive pressure wave equivalent to the modified water‐hammer pressure of a rain droplet at a velocity *V* = 150 m·s^−1^. This pressure is defined as $p_{wh}=\frac{V\rho_{l}c_{l}\rho_{c}c_{c}}{\rho_{l}c_{l}+\rho_{c}c_{c}}\;$, where ρ and *c* are the density and acoustic velocity of the liquid droplet (*l*) and the coating (*c*), respectively [41]. The amplitude of the reflected (σ_*R*_) and transmitted stress (σ_*T*_) in each interface is defined by the reflection and transmission coefficients $R=\frac{\sigma_{R}}{\sigma_{I}}\;$ and $T=\frac{\sigma_{T}}{\sigma_{I}}\;$, where σ_*I*_ is the incident stress amplitude [59]. For the first interface in the system, between the liquid droplet and the coating, this incident stress is equivalent to the water hammer pressure. Since the longitudinal (compressional) pulse primarily governs the peak stress during droplet impact [59], and the available 1‐D impedance relations are valid for this mode, the analysis focuses on this dominant stress component.

Two coating scenarios were analysed: a monolayer and a three‐layer gradient of the same thickness, both on top of a glass fibre‐epoxy substrate, as shown in Figure 4e. We performed calculations for platelet volume fractions of 0%, 1%, and 5% due to the validity of the time‐temperature superposition principle in the DMA results. This estimation neglects the through‐the‐thickness attenuation that will occur in each layer, which is expected to increase as the volume fraction decreases, given that the loss factor is higher in the relevant frequency regime [66]. In addition, the model treats the coating as a purely linear‐elastic, one‐dimensional wave propagation problem and does not incorporate a strain‐rate‐dependent strength or failure criterion. Since material strength typically increases substantially at the high strain rates associated with droplet impact, the calculated stresses exceeding quasi‐static strength values do not necessarily imply instantaneous damage on first impact, but rather reflect the idealized nature of the model, which is intended to isolate and compare the relative effect of impedance matching on the stress jump magnitude across layers. The initial water hammer pressure increases with platelet content due to the higher density and acoustic velocity, which in turn increases with the complex modulus. A higher acoustic velocity indicates the liquid is less compressible, so upon impact, it decelerates almost instantaneously. This abrupt deceleration transfers momentum more rapidly, generating a greater force and, consequently, a higher water‐hammer pressure [67]. For the monolayer scenario, the reflected stress at the coating‐substrate interface changes slightly for different volume fractions, with the initial water hammer pressure primarily governing this change.

For the gradient coating, we observe significantly lower reflected stresses at the different interfaces for a configuration with a 5% *V_f_* layer next to the substrate, a 1% *V_f_* middle layer, and a 0% *V_f_* top layer, compared to the inverse case with a 0% *V_f_* layer next to the substrate. This configuration achieves improved impedance matching between the coating and the substrate, resulting in lower reflected stresses and reducing the initial water hammer pressure due to the compliant outermost layer. Beyond mitigating reflections, this graded architecture also affects the stresses transmitted into the substrate. The progressive impedance transition across the layers allows the compressive stress wave to be gradually transmitted, lowering peak stress concentrations that might otherwise initiate damage mechanisms such as within the coating. This stress management strategy parallels natural impact‐resistant structures, where graded mechanical properties serve to absorb and redistribute energy efficiently, enhancing resilience under repeated erosive loading.

To validate the analytical predictions, we manufactured and tested the incubation time of a substrate–coating system by incorporating a 2.1 mm‐thick platelet‐reinforced polyurethane coating on a 40 mm × 40 mm × 4 mm glass fibre‐epoxy substrate, and tested its incubation time following the same erosion testing conditions as for the polyurethane‐platelet composites. We tested the monolayer with 0%, 1%, and 5% *V_f_*, and the gradient with three 0.7 mm‐thick sublayers of 0%‐1%‐5% *V_f_* and 5%‐1%‐0% *V_f_*, which will be referred to as S‐0‐1‐5 and S‐5‐1‐0 for simplicity. For the S‐0‐1‐5 gradient, the 0% *V_f_* sublayer is adjacent to the substrate and the opposite for the S‐5‐1‐0 gradient. Our choice of platelets over spheres was based on prior observations that in‐plane platelet orientation yields slightly superior erosion performance, as shown in Figure 2f. This advantage is hypothesized to be caused by the interaction of platelets with the Rayleigh wave front, which dominates surface stress propagation during droplet impact. Interestingly, functionalization of platelets to enable magnetic alignment, although effective for microstructural control, resulted in poorer erosion resistance without altering impedance, likely due to surface adhesion effects. Therefore, to avoid these effects, non‐functionalized platelets were selected. At these low concentration values, mechanical alignment with vibrating vortex equipment is sufficient to obtain in‐plane alignment [26]. The incubation times obtained for the monolayer systems, as shown in Figure 4f, are consistent with the previous results presented in Figure 2, where the erosion performance deteriorates with increasing platelet content. A different behavior is observed for the two gradients implemented, where the S‐0‐1‐5 coating presents a lower incubation time than the baseline case of no reinforcement. Conversely, the S‐5‐1‐0 coating exhibits the longest incubation time overall, with nearly double the incubation time of the following best‐performing case, as expected from the analytical estimations. The effect of impedance‐matched layers is evident from the damage at incubation time, where the S‐5‐1‐0 coating presents smaller and shallower damage compared to the S‐0‐1‐5 coating, in which full breakthrough of the coating was observed. The S‐5‐1‐0 gradient was designed for impact energy absorption with improved impedance matching near the substrate. It minimizes abrupt impedance changes between the coating and substrate while preserving the benefits of an outer soft layer, which reduces water hammer pressure and minimizes stress reflections from Rayleigh surface waves due to the absence of platelet‐polymer reflections. Furthermore, our gradient system minimizes adhesion issues commonly observed in multilayer coatings, as the gradient was achieved within a monolithic polyurethane matrix by gradually changing the platelet content.

Our proposed gradient minimizes interfacial adhesion penalties but raises a question about its compatibility with existing commercial leading‐edge protection systems. In such systems, a polyurethane‐based putty is applied over the substrate to enhance adhesion to the topcoat and introduce a partial acoustic transition at the substrate‐coating interface, which has the most critical impedance mismatch in the system. Our S‐5‐1‐0 architecture represents the optimized counterpart to this practice, introducing a better‐adhered multilayer system with a different physical configuration to improve acoustic transition. In a discrete multilayer stack, each interface will still produce partial wave reflections regardless of adhesion quality. In our proposed architecture, these reflections, although present due to the increased platelet content in the polyurethane, are progressively minimized as the gradient becomes smoother. The key advantage of this solution, therefore, persists even in the hypothetical absence of delaminations in a typical multilayered stack. The continuous impedance transition itself reduces stress wave reflections more effectively than a discrete‐step transition. Rather than replacing the putty later, whose geometric and adhesion‐promoting functions remain practically necessary, our gradient could be integrated with it to form a more complete and intentionally layered impedance‐graded system across the coating stack thickness. Whether such an integrated architecture yields further gains in incubation time compared to our current approach shows a meaningful direction for future experimental validation.

Our findings introduce a distinctive strategy within the broader landscape of reinforced coatings for leading‐edge erosion. While previous studies have demonstrated that particle reinforcement can enhance erosion resistance [17, 19, 20, 21, 22, 68], these approaches rely on uniform filler distributions or discrete multilayer stacks that often suffer from interfacial weaknesses and abrupt impedance transitions under rain erosion loading [11, 14, 59]. Recent studies have highlighted the importance of acoustic impedance matching and viscoelastic energy dissipation for mitigating water‐hammer stresses during droplet impact [7, 69]. Our design leverages these principles but advances the concept by implementing a monolithic gradient in platelet content, enabling impedance tailoring without introducing separate interfaces. This approach combines the damage control benefits of platelet reinforcement with a graded architecture inspired by biological systems, addressing both stress‐wave management and adhesion reliability.

## 3 Conclusions

A standalone alumina microplatelet‐reinforced polyurethane does not pose any significant enhancement for leading edge erosion protection. Rain erosion testing revealed a decrease in performance with increasing platelet addition, as well as the significant role of platelet orientation in damage morphology. We further demonstrated understanding of this behavior via Dynamic Mechanical Analysis in the relevant frequency regime, where a shift in the loss factor, from higher to lower values in the MHz regime, was observed for composites with platelet content above 5%. By clarifying the role of platelet content and orientation on rain‐induced wave propagation, damage morphology, and viscoelastic behavior, we propose a more efficient approach to implementing particle reinforcement on a polyurethane coating for leading edge erosion enhancement. We exploit this role and leverage the concept of impedance‐matched layers to reduce the reflection of stress waves that can harm both the coating and the substrate. Our work results in a bio‐inspired monolithic gradient coating with variable microplatelet content, which provides acoustic impedance matching closer to the substrate while preserving a soft outer layer that minimizes water hammer pressure. Manufacturing this gradient without functionalization or magnetic alignment also poses an advantage, as it can be compatible with current industrial application methods such as spray or roller coating, which naturally induce predominantly in‐plane orientations due to shear‐induced flow and gravitational settling. Beyond manufacturability, further gains in durability could be achieved by optimising particle‐matrix bonding and coating toughness alongside the impedance matching effect established here. This approach opens the way for further optimization of erosion‐resistant coatings through controlled microstructural design, enabling tailored wave mitigation strategies for high‐performance wind energy systems.

## 4 Experimental Section

### 4.1 Preparation of Functionalized, Magnetically‐Responsive Alumina Platelets

Aluminium oxide (Al_2_O_3_) platelets (White Sapphire, RonaFlair, Merck, Germany) with nominal diameter and thickness of 8.3 µm (D80< 25 µm) and 370 nm [26], respectively, were functionalized using an anionic aqueous ferrofluid (EMG‐705, Ferrotec, United States). This ferrofluid contains 12 nm superparamagnetic iron oxide nanoparticles (SPIONs) that coat the positively charged alumina platelets, thereby becoming magnetically responsive to an external magnetic field. The procedure to functionalize these platelets is described by Erb et al. [45]. An additional step was implemented to prevent the agglomeration of the platelets, where the functionalized platelets were mixed with deionized water in a closed glass container and subjected to an ultrasonic acetone bath (Emmi‐20HC, EMAG, Germany) for 1 h.

### 4.2 Preparation of Platelet‐Reinforced Polyurethane Composites

A commercial polyurethane with 60 Shore A hardness (Xencast PX60, Easy Composites, United Kingdom) was used to manufacture the platelet‐reinforced composites. The hard segments contain 4,4′‐methylene diphenyl diisocyanate, 3,5‐dimethylthio‐2,4‐toluenediamine, and 3,5‐dimethylthio‐2,6‐toluenediamine, while the soft segments consist of a prepolymer based on aromatic isocyanate. This polyurethane is available in two parts, part A and part B, which are mixed in a stoichiometric weight ratio of 1:1. Because the cured polyurethane has a translucent amber color, a white titanium oxide pigment (PEPU White Pigment, West & Senior, United Kingdom) was added in a 2% weight pigment‐to‐polyurethane ratio to facilitate visualization during erosion testing. In addition, a polyurethane thinner (PU‐Thinner, Vooschemie, Germany) was added in a 5% thinner‐to‐polyurethane weight ratio to facilitate molding. Both the pigment and thinner were added to all the samples to ensure the same viscosity. The mixture was then dried for 24 h at 150°C.

Monolithic composites with no alignment, in‐plane, and out‐of‐plane alignment were manufactured at platelet volume fractions of 0%, 0.5%, 1%, 5%, 10%, and 15%. The upper volume concentration was limited by the effective viscosity of the polyurethane‐alumina suspension, as casting was not possible above a volume fraction of 15%. Before mixing any components, both parts of the polyurethane were degassed separately for 30 min. First, the pigment and thinner were mixed in a speedmixer (DAC 400.2 VAC‐P LR, Hauschild, Germany) at a rotational speed of 2000 rpm. Part A of the polyurethane and the functionalized platelets were added, and the solution was mixed and degassed for 3 and 8 min, respectively. Finally, Part B of the polyurethane was added, and the final solution was mixed for 1 min. The uncured polyurethane‐platelet suspension was cast in a silicone mold (dimensions vary depending on the test) and subjected to a rotating magnetic field of 130 mT at 10 Hz for 15 min. This field was generated by a rotating magnet (Death Magnet, Supermagnete, Germany) attached to a drill (RW28 digital, IKA, Germany). The magnetic field was calibrated using a magnetic field analyser (MF100, EXTECH, UK), and the rotational speed was measured with a digital tachometer (DT‐10L, Volcraft, Germany). To facilitate platelet orientation, the mold was vibrated with a vortex mixer (VV3, IKA, Germany) at 5000 rpm. The chosen frequency of rotation corresponds to the critical value at which the platelet orientation is limited to the plane of the rotating magnetic field by the viscous forces exerted by the uncured polyurethane [70]. This frequency depends on the polymer viscosity, platelet dimensions, external magnetic field, and SPIONs magnetization [45]. Further information on the calculation of the critical frequency can be found in Figures S14 and S15.

### 4.3 Preparation of Sphere‐Reinforced Polyurethane Composites

Aluminium oxide (Al_2_O_3_) spheres with a nominal diameter of 10 µm (BAK‐10, Xtra Advanced Materials, Germany) were used to manufacture the composites with spherical reinforcement. These spheres were not functionalized as magnetization was not necessary. They were dried in the oven for 24 h at 150°C to remove any moisture. To manufacture composites with volume fractions of 1%, 5%, and 15%, the platelets were mixed with the PX60 polyurethane system using the same procedure described for the platelets.

### 4.4 Preparation of Glass Fibre Substrate with a Polyurethane‐Platelet Coating

Glass fibre‐epoxy composite laminates were manufactured using vacuum‐assisted resin infusion. Six ± 45 dry glass plies (X‐E‐810 g/m^2^–1270 mm, Saertex, Germany) were laid onto a flat steel mold, covered with a peel ply, perforated release film, flow mesh, and vacuum bagging film. After applying full vacuum, the resin (EPIKOTE‐EPIKURE Resin System 04908, Hexion, United States) was infused and allowed to cure for 24 h. The laminate had a nominal thickness of 3.98 mm and was cut into 40 mm ×  40 mm specimens. The mold side of the specimens was sanded with 800‐grit paper to improve adhesion with the polyurethane suspension. Using a 3D‐printed jig, the polyurethane‐alumina suspension was cast over the substrate to ensure a thickness of 2 mm. After casting, the stack was vibrated with a vortex mixer (VV3, IKA, Germany) at 5000 rpm to mechanically align the platelets in‐plane, as this has been proven to be sufficient for in‐plane orientation at low volume fractions [26]. For samples with a gradient coating, each layer was cast and allowed to cure for 10 min, right before the end of the working time, prior to applying the next layer. This time was enough to constrain the platelet motion in each layer while allowing the polyurethane to adhere properly between layers, therefore obtaining a monolithic polyurethane coating.

### 4.5 Erosion Testing

Rain erosion experiments were conducted using a Pulsating Jet Erosion Tester (PJET, Ducom Instruments, India), as described in the study of Verma et al. [29]. Flat samples with nominal dimensions of 40  × 40 ×  2.5 mm^3^ were mounted normal to the jet axis, and water jets were generated through a 1.5 mm nozzle. For each sample, up to four erosion tests were performed due to a minimum distance requirement of 1 cm between the point of impact and the sample edge, and 2 cm between each point of impact. The continuous jet stream is cut with a rotating disc containing two 2‐mm holes to produce discrete water slugs, which have an approximate diameter of 2 mm. The impact velocity was set to 170 m s^−^^1^, and the impact frequency was fixed at 27.5 Hz. Tests were performed without dry intervals to represent continuous exposure. 3 h prior to testing, the PJET was turned on to allow the water to stabilise at a temperature of 38°C, and 48 h prior to testing, the specimens were put in an environmental chamber (WK111 340, Weiss, Germany) at 38°C and 98% relative humidity to simulate testing conditions. Each impact point in the sample was tested for a specific amount of time to study the erosion rate, and the incubation time (the time when damage is first visible) was recorded from outside the testing chamber. The incubation time was recorded three times per specimen.

### 4.6 Microscopy

Surface topography and erosion damage were characterized using a wide‐area 3D microscope (VR‐5000, Keyence, Japan). The microscope uses light projection to capture stitched height changes over large surface areas, allowing the measurement of eroded volume and surface morphology. The height images were later processed in MATLAB to convert the color scheme to a global scale for all images and produce the erosion damage maps.

### 4.7 Dynamical Mechanical Analysis of Particle‐Reinforced Composites

Composites with nominal dimensions of 1.25 × 6 ×  40 mm^3^ were tested on an RSA G2 DMA equipment (TA Instruments, United States) using a rectangular tension jig. The specimens were clamped with a torque of 15 cN.m and preloaded with a force of 0.1 N to ensure tensile loading. Temperature sweeps were performed on a temperature range of −50°C to 40°C with a soaking time of 10 min. At each 10°C step in the sweep, the specimen was scanned over a frequency range of 0.1 to 100 Hz with an oscillatory amplitude of 10 µm. From these measurements, the storage modulus (*E′*), loss modulus (*E″*), and loss factor (*tanδ)* were obtained. These sets of data were sufficient to implement a time‐temperature superposition (TTS) to extend the frequency range. This TTS master curve was created using the TRIOS software from TA Instruments. More information on the shift factors can be found in Figure S12.

The neat polyurethane (0% V_f_), which was tested three times to confirm measurement repeatability. Then each composite formulation was tested once per temperature to generate the master curve. For those formulations, repeatability was assessed through the construction of the TTS master curve. Since each temperature step is measured independently and then shifted along the frequency axis, a smooth and continuous master curve, free of discontinuities between adjacent temperature segments, suggests that the underlying single measurements are consistent. At a given platelet concentration, samples with different platelet orientations showed no discernible difference in storage modulus, loss modulus, or loss factor, providing further evidence supporting measurement repeatability.

The validity of the TTS‐estimated data based on the criteria suggested by TA Instruments (presented in Figures S10–S12) was confirmed for composites with no alignment and 0%, 0.5%, 1%, and 5% platelet volume fraction. However, for composites with 10% and 15% platelet volume fractions, at least one criterion was not valid; therefore, the TTS results may not be applicable. This might occur because adding platelets introduces new interactions between the polyurethane segments and platelets, which is a core assumption in the TTS principle, thereby eliminating the thermorheological simplicity of the material. These effects become more relevant at higher platelet volume fractions, explaining why the validation criteria fail after a certain volume fraction. For this reason, we present the results with a clear distinction between what was measured and what was estimated.

### 4.8 Dynamical Mechanical Analysis of Nacre

A nacre sample (Mother of Pearl White 2 mm, Fischer, Germany) of dimensions 2 × 6 ×  40 mm^3^ was tested on an RSA G2 DMA equipment (TA Instruments, United States) using a three‐point bending jig. Temperature sweeps were performed over a temperature range of −30°C to 80°C with a soaking time of 10 min. At each 10°C step in the sweep, the specimen was scanned over a frequency range of 0.1 to 100 Hz with an oscillatory amplitude of 40 µm. From these measurements, the storage modulus (*E′*), loss modulus (*E″*), and loss factor (*tanδ)* were obtained (Figure S4).

### 4.9 Viscosity Measurements

The viscosity of the polyurethane with thinner and pigment was measured using a rotational rheometer (Haake Mars III, Thermo Scientific, United States) with a 20‐mm diameter flat aluminium plate and a 1‐mm gap. A time sweep was performed at a constant shear rate of 1 s^−^^1^ over a period of 10 min at room temperature. These viscosity values were used to determine the critical frequency of the external rotating magnetic field (Figures S14 and S15).

### 4.10 SEM

A scanning electron microscope (JSM‐7500F, JEOL, The Netherlands) operated with an acceleration voltage of 5 kV was used to visualise the microstructure of the nacre‐like composites. These composites were previously coated with a 15‐nm gold film using a plasma sputter (Quorum Q300TD, United Kingdom).

### 4.11 High‐Speed Camera

A Photron FASTCAM NOVA S Series high‐speed camera was used to record the droplet impact in slow motion. A rate of 1000 frames per second and a shutter speed of 0.1 µs were shown to be sufficient to capture the required phenomena.

## Author Contributions

**Natalia Sofia Guevara‐Sotelo**: investigation. **All**: conceptualization, methodology, visualization, formal analysis, validation. **Julie Teuwen** & **Kunal Masania**: supervision. **Julie Teuwen** & **Kunal Masania**: funding acquisition. **All**: writing – original draft. **All**: writing – review and editing.

## Conflicts of Interest

The authors declare no conflicts of interest.

## Supporting information




**Supporting File**: advs77056‐sup‐0001‐SuppMat.docx.

## Data Availability

The data supporting the findings of this study is available from the corresponding author upon reasonable request.
