# Supplementary material for: Bio‐Inspired Gradient Coatings Enhance the Leading Edge Erosion Resistance of Wind Blades
Source: Adv Sci (Weinh). 2026 Aug 6;13(60):e77056. doi: 10.1002/advs.77056 (PMC13445328; doi:10.1002/advs.77056)
Supplement: Supplementary file 1 [file ADVS-13-e77056-s001.docx]

**SUPPORTING INFORMATION:**

**Development of a bio-inspired alumina-platelet gradient coating for enhancing the leading edge erosion resistance of wind turbine blades**

N.S. Guevara-Sotelo^1^, J. J. E. Teuwen^1,*^, K. Masania^1,*^

^1^Faculty of Aerospace Engineering, Delft University of Technology, Kluyverweg 1, 2629 HS Delft, Netherlands

*Corresponding authors: [J.J.E.Teuwen@tudelft.nl](mailto:J.J.E.Teuwen@tudelft.nl) , [K.Masania@tudelft.nl](mailto:K.Masania@tudelft.nl)

**Figure S1.** The erosion maps show the damage evolution over jet impact time for composites with no aligned platelets. The damage images were obtained using a wide-area microscope, which allows for the measurement of both area and depth.

**Figure S2.** The erosion maps show the damage evolution over jet impact time for composites with in-plane aligned platelets. The damage images were obtained using a wide-area microscope, which allows for the measurement of both area and depth.

**Figure S3.** The erosion maps show the damage evolution over time for jet impact on composites with out-of-plane aligned platelets. The damage images were obtained using a wide-area microscope, which allows for the measurement of both area and depth.

**Figure S4.** The plots display the storage modulus, loss modulus, and loss factor (tan δ) of a natural nacre sample over a temperature range of -30 °C to 80 °C, with 10 °C increments.

**Figure S5.** The plots display the storage modulus, loss modulus, and loss factor for platelet-reinforced composites with **in-plane alignment**. A change in the behaviour of the loss factor is observed around the estimated natural frequency of the neat polyurethane (343 Hz).

**Figure S6.** The plots display the storage modulus, loss modulus, and loss factor for platelet-reinforced composites with **out-of-plane alignment 1**. A change in the behaviour of the loss factor is observed around the estimated natural frequency of the neat polyurethane (343 Hz).

**Figure S7.** The plots display the storage modulus, loss modulus, and loss factor for platelet-reinforced composites with **out-of-plane alignment 2**. A change in the behaviour of the loss factor is observed around the estimated natural frequency of the neat polyurethane (343 Hz).

**Figure S8.** The plots display the storage modulus, loss modulus, and loss factor for **sphere-reinforced** composites. A change in the behaviour of the loss factor is not observed around the estimated natural frequency of the neat polyurethane (343 Hz).

**Figure S9.** The plots display the storage modulus, loss modulus, and loss factor for **non-functionalised** platelet-reinforced composites. A change in the behaviour of the loss factor is observed around the estimated natural frequency of the neat polyurethane (343 Hz).

The manufacturer of the DMA Solids Analyzer (TA Instruments, United States) states that the validity of the TTS can be determined if all of the following conditions are met [2]:

1. Van-Gurp Palmen plot (Phase angle (δ) vs. Complex modulus (*E**)) should be a smooth curve
2. Cole-Cole plot (Loss modulus (*E’’*) vs. Storage modulus (*E’*)) should be a smooth curve
3. Shift factor should fit the Williams–Landel–Ferry (WLF) or Arrhenius equation. For this TTS, WLF was used:

$$\log\left( a_{T} \right)=\frac{-C_{1}(T-T_{o})}{C_{2}+T-T_{o}}$$

Where $a_{T}$ is the horizontal shift factor, $T$ is the temperature, $T_{o}$ is the reference temperature (30 °C), and $C_{1} \& C_{2}$ are curve fitting constants.

4. The same shift factor should be applicable to all viscoelastic parameters. The TRIOS software of TA Instruments automatically sets this condition, as it uses the same factors to shift all the master curves (*E’, E’’, tan(δ)*).

Table S1 shows the conditions that were met for each platelet volume fraction. From these, 10% and 15% *V_f_* did not meet any of the requirements, and their validity cannot be confirmed. Figures S12 to S14 show the plots used to determine whether the conditions were met.

**Table S1.** Summary of the conditions to validate the TTS of the platelet-reinforced composites with varying platelet content.

| *V_f_* (%) | Condition 1 | Condition 2 | Condition 3 | Condition 4 |
| --- | --- | --- | --- | --- |
| 0 | ✓ | ✓ | ✓ | ✓ |
| 0.5 | ✓ | ✓ | ✓ | ✓ |
| 1 | ✓ | ✓ | ✓ | ✓ |
| 5 | ✓ | ✓ | ✓ | ✓ |
| 10 | × | × | × | × |
| 15 | × | × | × | × |

**Figure S10.** Van-Gurp Palmen plots (Phase angle (δ) vs. Complex modulus (*E**)) of composites with platelet volume fraction 0% - 15% and no aligned platelets. These plots were used to validate condition 1. Composites with *V_f_* ranging from 0% to 5% present a smooth curve, whereas this is not the case for *V_f_* values of 10% and 15%.

**Figure S11.** Cole-Cole plots (Loss modulus (*E’’*) vs. Storage modulus (*E’*)) of composites with platelet volume fraction 0% - 15% and no aligned platelets. These plots were used to validate condition 2. Composites with *V_f_* ranging from 0% to 5% present a smooth curve, whereas this is not the case for *Vf* values of 10% and 15%.

**Figure S12.** Horizontal shift factor plots for each temperature data set used to create the master curves (*E’, E’’, tan(δ)*). Condition 3 is met if the shift factors fit the WLF model, which was the case for composites with 0% to 5% *V_f_*.

Table S2 shows the$C_{1}$, $C_{2}$ parameters and R^2^ values of the WLF fitting. obtained from Williams–Landel–Ferry (WLF) fits to horizontal shift-factor data.

**Table S2.** Parameters of the Williams–Landel–Ferry fitting of the platelet-reinforced composites with varying platelet content.

| ***V_f_ (%)*** | **C_1_** | **C_2_** | **R^2^** |
| --- | --- | --- | --- |
| 0 | 9.29 | 172.76 | 0.999 |
| 0.5 | 5.45 | 132.55 | 0.994 |
| 1 | 9.31 | 168.20 | 0.999 |
| 5 | 15.28 | 219.64 | 0.999 |
| 10 | - | - | - |
| 15 | - | - | - |

**Figure S13.** The plot displays the time-temperature superposition of the Dynamic Mechanical Analysis of the alternative polyurethane system with 90 Shore A hardness (Xencast PX90, Easy Composites, United Kingdom). A change in the behaviour of the loss factor is observed around the estimated natural frequency of the neat polyurethane.

The composites were manufactured following the same procedure as described for the PX60 in the experimental section, but using a harder polyurethane with 90 Shore A hardness (Xencast PX90, Easy Composites, United Kingdom).

**Figure S14.** The plot displays the evolution of viscosity over time for three systems: mixed polyurethane, mixed polyurethane with pigment, and mixed polyurethane with pigment and thinner. The effect of adding pigment and thinner to the polyurethane on its viscosity needs to be determined to find the critical frequency at which the external magnetic field must rotate.

**Figure S15**. The plot displays the viscosity evolution of the mixed polyurethane with pigment, and thinner over time and the effect it has on the critical frequency [1] of the rotating external magnetic field.

**References**

[1]	R. M. Erb, J. Segmehl, M. Schaffner, and A. R. Studart, “Temporal response of magnetically labeled platelets under dynamic magnetic fields,” *Soft Matter*, vol. 9, no. 2, pp. 498–505, 2013, doi: 10.1039/C2SM26910D.

[2]	“Online Training Course Downloads - TA Instruments.” Accessed: Oct. 21, 2025. [Online]. Available: https://www.tainstruments.com/online-training-course-downloads/
